# CRISPR activation: identifying and using novel genes for plant disease resistance breeding

**DOI:** 10.3389/fgeed.2025.1596600

**Published:** 2025-09-08

**Authors:** John E. McLaughlin, Idrice Carther Kue Foka, Michael A. Lawton, Rong Di

**Affiliations:** Department of Plant Biology, Rutgers University, New Brunswick, NJ, United States

**Keywords:** CRISPR activation, CRISPR/dCas9, activation tagging, genome-wide association studies, plant disease resistance, gain-of-function, programmable transcriptional activators CRISPR activation, programmable transcriptional activators

## Abstract

CRISPR-based technologies have revolutionized plant science by enabling precise modulation of gene function, including CRISPR activation (CRISPRa), a recently emerging strategy which shows particular promise for enhancing disease resistance through targeted gene upregulation. Unlike conventional CRISPR editing, which introduces double-stranded DNA breaks and permanent genomic changes, CRISPRa employs a deactivated Cas9 (dCas9) fused to transcriptional activators. This system allows quantitative and reversible gene activation without altering the DNA sequence, offering a gain-of-function (GOF) like enhanced blight resistance in staple crops. Despite its potential, the limited adoption of CRISPRa in plant biology to date underscores the need for future studies to fully harness its capabilities for crop improvement. This review addresses the groundbreaking and relatively underexplored potential of CRISPR activation (CRISPRa) systems for GOF studies in plant biology, and advocates for the adoption of CRISPRa to discover and harness genetic variation for enhancing disease resistance. We present recent advancements in CRISPRa technology, emphasizing its successful application in boosting plant immunity. Moreover, we discuss the synergistic potential of integrating CRISPRa with functional genomics tools such as genome-wide association studies (GWAS) and multi-omics approaches to identify and characterize key resistance genes. Additionally, we highlight ongoing progress in developing plant-specific programmable transcriptional activators (PTAs) to optimize CRISPRa efficiency. Challenges associated with achieving transgene-free overexpression and the deployment of alternative CRISPR systems are also explored. Together, these advances position CRISPRa as a transformative tool for future crop breeding strategies aimed at achieving durable, broad-spectrum disease resistance and sustainability in agriculture.

## Introduction

In the face of increasing pathogen pressure and climatic variability, safeguarding crop productivity is a critical global challenge (Stukenbrock and Gurr, 2023; Nelson et al., 2018; Donatelli et al., 2017). The increasing demand for agricultural productivity, driven by global population growth and climate change, necessitates the development of crops with enhanced resistance to both biotic and abiotic stresses (Roberts and Mattoo, 2018; Gonzalez Guzman et al., 2022). To address this challenge, advanced plant breeding methodologies are continually being developed and refined to help accelerate genetic gains and improve crop resilience. Traditional approaches, including mutational techniques such as ethyl methane sulfonate (EMS) and ionizing radiation, have been instrumental in generating novel genetic variations for selection (Ma et al., 2021; Oladosu et al., 2016). Genetic modification via transgene insertion has also proven effective, exemplified by traits like herbicide resistance and enhanced β-carotene content in golden rice (Beyer et al., 2002; De Block et al., 1987). However, these methods may induce random, untargeted mutations across the entire genome, often requiring extensive screening to identify desired traits and frequently leading to unintended pleiotropic effects caused by gene silencing, or the disruption of endogenous genes.

The advent of genome editing technologies, particularly CRISPR (Clustered Regularly Interspaced Short Palindromic Repeats)/Cas, has revolutionized the precision and efficiency of genetic modification (Gonzalez Guzman et al., 2022; Arora and Narula, 2017; Doudna and Charpentier, 2014; Berman et al., 2025). Originally characterized as a bacterial adaptive immune system, CRISPR has emerged as a groundbreaking tool for targeted gene editing, allowing researchers to introduce specific modifications that can generate gene knockouts, cause beneficial mutations, or fine-tuned gene expression (Ali et al., 2023; Boubakri, 2023; Dhugga, 2022; Wang and Doudna, 2023). Its application has already led to improved crop traits, including enhanced disease resistance, drought tolerance, and improved nutritional profiles (Low et al., 2019; Zhong et al., 2022; Li et al., 2021). Initial research on CRISPR technology was primarily conducted on *Arabidopsis thaliana*, a well-established model plant with genetic attributes conducive to experimental studies. *Arabidopsis* played a crucial role in the foundational development and refinement of CRISPR-based genome editing tools, including optimizing gene targeting efficiency and vector design (Miki et al., 2018). This early and ongoing work in *Arabidopsis* paved the way for the efficient translation of CRISPR technologies to agriculturally important crops.

While most functional genomic studies have relied on the induction and study of loss-of-function (LOF) mutations, gain-of-function (GOF) approaches offer unique insights, especially when gene redundancy obscures phenotypes (Casadevall et al., 2024; Saalbach, 2022). GOF mutations can be achieved through methods like activation tagging (Gou and Li, 2012), transgene overexpression (Karunadasa et al., 2022), or targeted gene editing to produce hyperactive variants, thereby providing valuable insights into the functional role of genes, particularly when studying gene families with functional redundancy. In such cases, gene knockouts may fail to reveal phenotypic changes due to compensation by homologous genes (Rossi et al., 2015; El-Brolosy and Stainier, 2017). Recent advancements in CRISPR technology have made it possible to employ CRISPRa to generate GOF mutations (Pan et al., 2021a; Heidersbach et al., 2023). CRISPRa primarily utilizes a dCas9 protein fused with transcriptional activators to upregulate the gene target’s expression without altering its DNA sequence (Yao et al., 2023; Pan et al., 2022). This precise, targeted approach offers significant advantages over traditional methods of random mutagenesis or transgene-based overexpression. Transgene-based overexpression, which usually involves the random insertion of foreign DNA sequences can suffer from unpredictable positional effects. In contrast, CRISPRa activates endogenous genes in their native genomic context, thereby minimizing off-target effects and preserving the integrity of the plant genome (Touzdjian Pinheiro Kohlrausch Távora et al., 2022).

The ability of CRISPRa to fine-tune gene expression can be used to elucidate gene functions that would otherwise remain undetected. For example, GOF screens have successfully identified genes conferring stress tolerance, thus providing new opportunities for enhancing crop resilience through genetic manipulation (Yang et al., 2024; Benslimane, 2020; McLaughlin et al., 2015). This precision also enables researchers to systematically and rapidly test and validate candidate genes for their role in enhancing desirable traits such as plant development, disease, and abiotic stress resistance. For instance, CRISPRa was successfully employed to epigenetically reprogram the *SlWRKY29* gene in the Micro-Tom tomato, a model tomato developed for scientific research. This approach established a transcriptionally permissive chromatin state that enhanced somatic embryo induction and maturation and which has great significance for improved crop trait development (Valencia-Lozano et al., 2024; Shikata and Ezura, 2016). CRISPRa has also been used to successfully enhance tomato plant defense against *Clavibacter michiganensis* infection by upregulating the *PATHOGENESIS-RELATED GENE 1* (*SlPR-1*) (García-Murillo et al., 2023) and by upregulating the *SlPAL2* gene through targeted epigenetic modifications, leading to enhanced lignin accumulation and increased defense (Rivera-Toro et al., 2025). Recently, a CRISPR–dCas9–6×TAL-2×VP64 (TV) system was successfully employed in *Phaseolus vulgaris* hairy roots to upregulate defense genes encoding the antimicrobial peptides *PvD1*, *Pv-thionin*, and *Pv-lectin* using. This approach resulted in significant increases in target gene expression (e.g., 6.97-fold for *Pv*-lectin) (Maximiano et al., 2025). The integration of CRISPRa with other functional genomics approaches, such as GWAS and multi-omics data, holds tremendous potential for accelerating the discovery of novel resistance genes (Jamil et al., 2025). Additionally, the development of plant-specific programmable transcriptional activators (PTAs) is expected to further enhance the selectivity and utility of CRISPRa in crop improvement (Bikard et al., 2013; Casas-Mollano et al., 2023; Jinek et al., 2012).

Despite these advancements, several challenges remain for the widespread adoption and optimization of CRISPRa. Achieving transgene-free overexpression, optimizing CRISPRa systems for diverse plant species, and implementing alternative CRISPR systems all require further investigation and development. Nevertheless, the promise of CRISPRa as a tool for harnessing GOF mutations to enhance disease resistance and other desirable traits in crops is already apparent (Nidhi et al., 2021; Barrangou and Doudna, 2016). This review provides a comprehensive overview of the current state of GOF mutagenesis in crop improvement, focusing particularly on its application in enhancing plant disease resistance. We highlight the strengths and limitations of various screening methodologies and present compelling examples of successful CRISPRa applications in elucidating gene function. By focusing on CRISPRa as a tool for activating endogenous defense genes, this review explores an underutilized strategy for building disease-resistant crops.

## General background on CRISPR

The CRISPR/Cas system, originally characterized as a bacterial adaptive immune mechanism against invading viruses, has revolutionized genetic engineering due to its remarkable efficiency, precision, and versatility (Wang and Doudna, 2023; Barrangou and Marraffini, 2014; Chen et al., 2019). Among the various CRISPR systems, the type II CRISPR-Cas9 has emerged as a powerful tool for targeted genome editing, finding application in both fundamental research and agricultural biotechnology (Liu et al., 2017; Liang et al., 2016). The CRISPR-Cas9 system comprises a Cas9 nuclease guided by a dual RNA complex, consisting of a CRISPR RNA (crRNA) hybridized with a trans-activating crRNA (tracrRNA). To simplify this system, researchers fused the crRNA and tracrRNA into a single guide RNA (sgRNA), thereby enhancing its utility for genome editing. CRISPR target site recognition requires a protospacer-adjacent motif (PAM) sequence, typically NGG in the case of *Streptococcus pyogenes* Cas9 (Le Rhun et al., 2019; Steinert et al., 2015). During the editing process, the guide RNA forms an RNA-DNA heteroduplex with the complementary DNA strand, guiding Cas9 to introduce a double-stranded break (DSB) through its RuvC and HNH nuclease domains. The DSB can then be repaired by either non-homologous end joining (NHEJ) or homology-directed repair (HDR) (Yang et al., 2020; Zaboikin et al., 2017).

**FIGURE 1:**
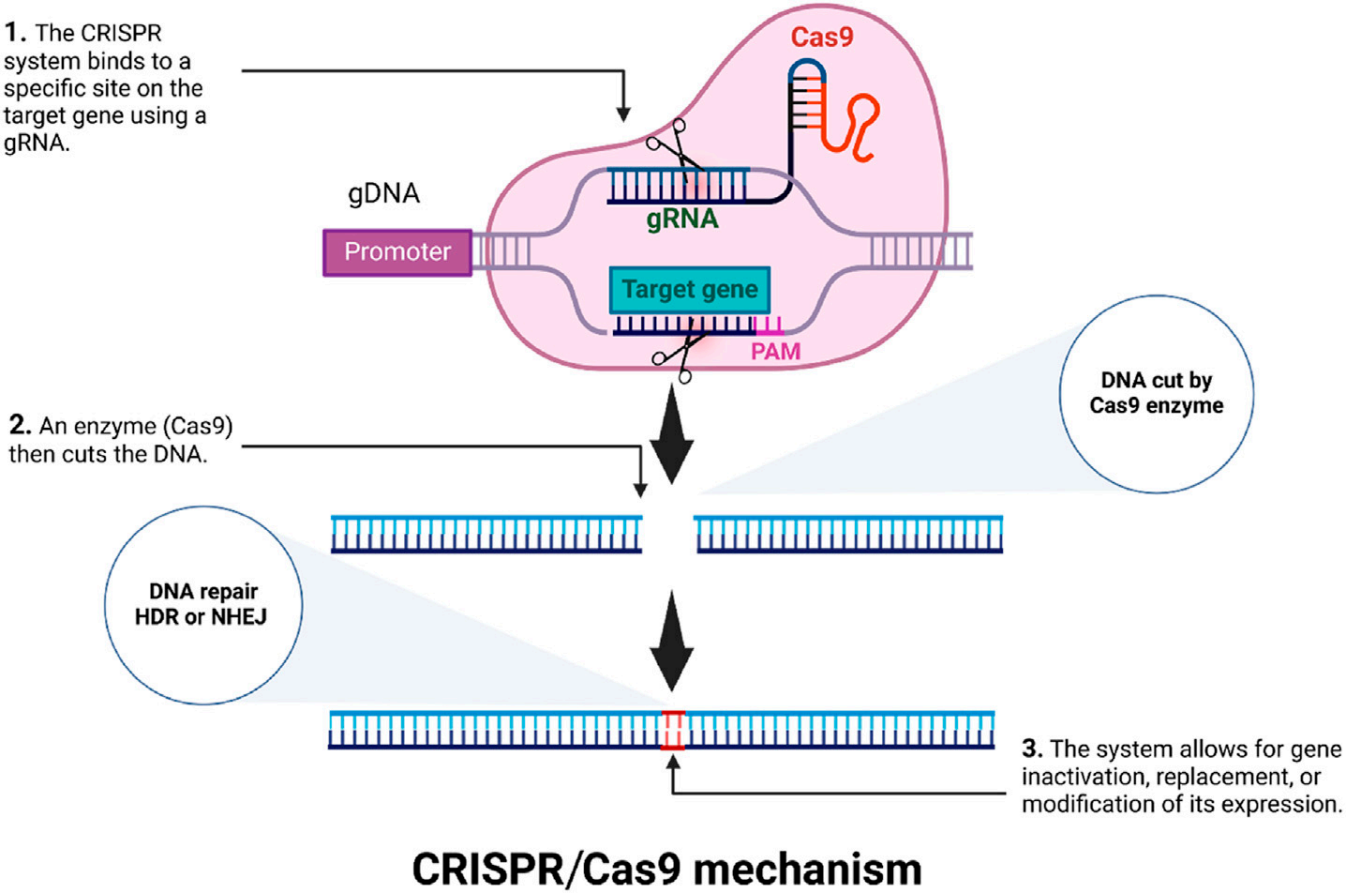
Diagram of the CRISPR-Cas9 system.

NHEJ, which is the more prevalent repair mechanism, often introduces insertions or deletions (indels) that can disrupt gene function through frameshift mutations, effectively creating knockouts (Figure 1). This highly efficient and scalable mutagenesis approach has dramatically accelerated functional genomics studies, enabling large scale genotype-phenotype analyses to be performed (Maruyama et al., 2015; Molla et al., 2022). Additionally, the development of base editing, an adaptation of CRISPR-based approaches, allows precise point mutations to be made without relying on HDR or donor DNA templates (Miki et al., 2018). Base editors are typically composed of a Cas9 nickase (Cas9n), with an inactivated RuvC domain, fused to a DNA deaminase enzyme. Two primary classes of base editors have been established: cytosine base editors (CBEs), which convert C-G base pairs to T-A, and adenine base editors (ABEs), which mediate A-T to G-C transitions (Li et al., 2023; Azameti and Dauda, 2021). The development of these tools for precise genome modification has greatly broadened the opportunities for manipulating crop genomes and traits.

The utility of CRISPR-Cas9 in crop improvement has been demonstrated in various studies. For instance, in 2020, CRISPR-Cas9 was employed to disrupt the *OsProDH* gene in rice, resulting in increased proline accumulation, reduced reactive oxygen species levels, and enhanced thermotolerance (Rossi et al., 2015; Guo et al., 2020). Similarly, CRISPR-Cas9 was used to study the role of polygalacturonase in cell wall immune responses by targeting the *OsPG1* gene, thereby elucidating the importance of cell wall integrity in bacterial resistance (Cao et al., 2021). CRISPR-Cas9 technology has also shown high mutation efficiency across diverse crops. Its first reported application in soybean involved the knockout of the green fluorescent protein (GFP) gene, paving the way for numerous studies targeting agronomically important traits (Jacobs et al., 2015). Importantly, multiplex CRISPR-Cas9 approaches can be employed to simultaneously create more than one mutation in eukaryote cells, making the technology more versatile and efficient. For example, multiplex CRISPR-Cas9 was used to create triple knockouts of *GmF3H1*, *GmF3H2*, and *GmFNSII-1*, leading to increased isoflavone content and enhanced resistance to soybean mosaic virus (SMV) (Zhang et al., 2020).

Our own previous research applied CRISPR technology to knock out two susceptibility genes involved in *Fusarium graminearum* infection in *Arabidopsis thaliana*, demonstrating the potential to enhance resistance to *Fusarium* head blight (FHB) in barley (Low et al., 2020; Low et al., 2022). CRISPR knocking out the *homoserine kinase* gene in sweet basil (*Ocimum basilicum*), successfully produced transgene-free, downy mildew-resistant mutant plants (Zhang et al., 2021). The application of CRISPR/Cas technology in managing biotic stresses, including pathogens such as bacteria, viruses, fungi and pests has been widely explored. One approach involves targeting susceptibility (S) genes that pathogens exploit to facilitate infection (van Schie and Takken, 2014; Zaidi et al., 2018). By knocking out these genes, infection and spread of plant disease can be significantly attenuated. For instance, the disruption of *OsSWEET14* in rice conferred resistance to *Xanthomonas oryzae*, the causative agent of bacterial blight (Zeng et al., 2020). Similarly, the mutation of the *MLO* gene in wheat resulted in improved resistance to powdery mildew (Zhang et al., 2021; Ingvardsen et al., 2019). CRISPR technology has also been applied to directly target pathogen genomes. For example, the use of CRISPR-Cas9 to disrupt geminivirus genomes in plant cells has shown promise in reducing viral replication and disease severity (Zeng et al., 2020; Sh et al., 2023). Moreover, significant progress has been made in utilizing CRISPR-Cas9 genome editing technology to disrupt gene function in filamentous fungi, offering a versatile and efficient approach for functional genomics studies (Louwen et al., 2014; Gosavi et al., 2020; Song et al., 2019).

### GOF mutants: activation tagging and CRISPRa

#### Activation tagging

**FIGURE 2:**
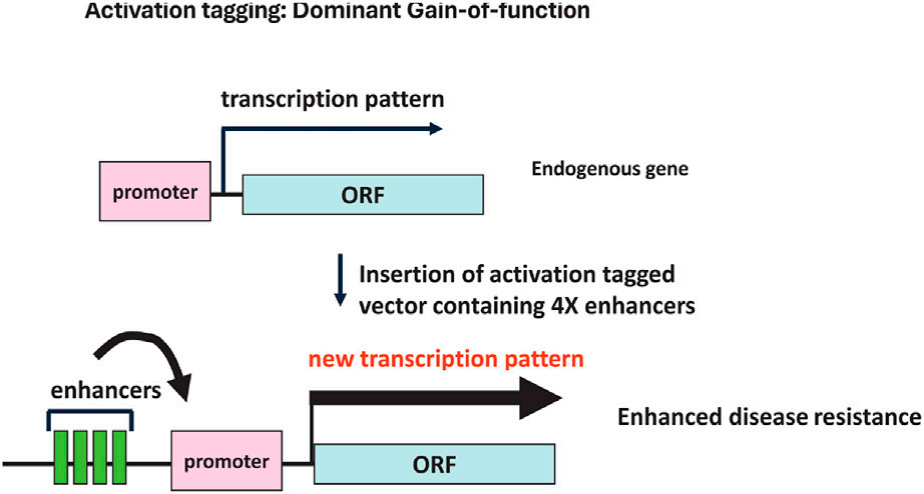
Activation tagging schematic diagram.

Insertional mutagenesis, as a functional genomics approach in plant genetics, has been a powerful tool for studying gene function (Bouchez and Höfte, 1998; Przybyla and Gilbert, 2022; Ayliffe and Pryor, 2009). Traditionally, this has involved the random insertion of genetic elements, such as T-DNA or the maize transposon system (AC/DS), into the plant genome. When these elements carry strong enhancers or promoters, their insertion near a gene can lead to overexpression of that gene (Weigel et al., 2000; Fladung, 2016). An example of this change in transcription pattern due to the insertion of enhancers is presented in Figure 2. This method, known as activation tagging, generates GOF mutants, which are instrumental in uncovering the roles of genes that might otherwise remain functionally hidden, due to redundancy or whose precise role in development may be obscured by the lethality of LOF mutations. Activation tagging has enabled the identification of numerous genes involved in various plant processes, including stress responses, growth regulation, and developmental pathways (Ayliffe and Pryor, 2009; Fladung, 2016; Dutta et al., 2021; Mahendranath et al., 2023; Wan et al., 2009). Examples of successful activation tagging applications include the identification of leaf and fruit color mutants in tomato, such as the anthocyanin color1 (*ant1*) mutant, which exhibits a significant accumulation of anthocyanins due to the overexpression of a MYB transcription factor regulating anthocyanidin biosynthesis (Mathews et al., 2003). The development of the Purple tomato, a cherry tomato brought to market in 2024 by Norfolk Plant Sciences, traces its success back to activation tagging and the identification of transcription factors that control anthocyanidin biosynthesis (Zhi et al., 2020). Disease resistance genes have also been identified using this technique, with notable discoveries including genes conferring resistance to downy mildew in *A. thaliana* (Gao et al., 2014; Grant et al., 2003), bacterial blight and sheath blight resistance in rice (Gandikota et al., 2024; Vo et al., 2018; Mori et al., 2007) and trichothecene and FHB resistance in *Arabidopsis* and wheat (McLaughlin et al., 2015; McLaughlin et al., 2021).

### CRISPRa in functional genomics: advancing plant immunity

**FIGURE 3:**
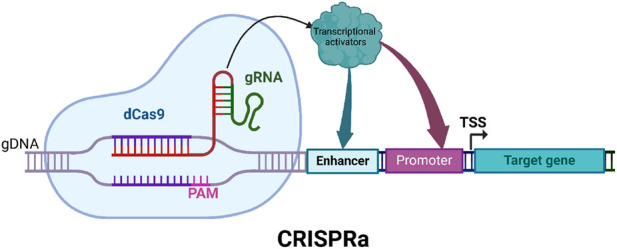
Illustration of CRISPR/dCas9-mediated transcriptional activation. The dCas9 domain is fused to transcriptional activators to activate adjacent promoters and the transcription of associated genes.

**FIGURE 4:**
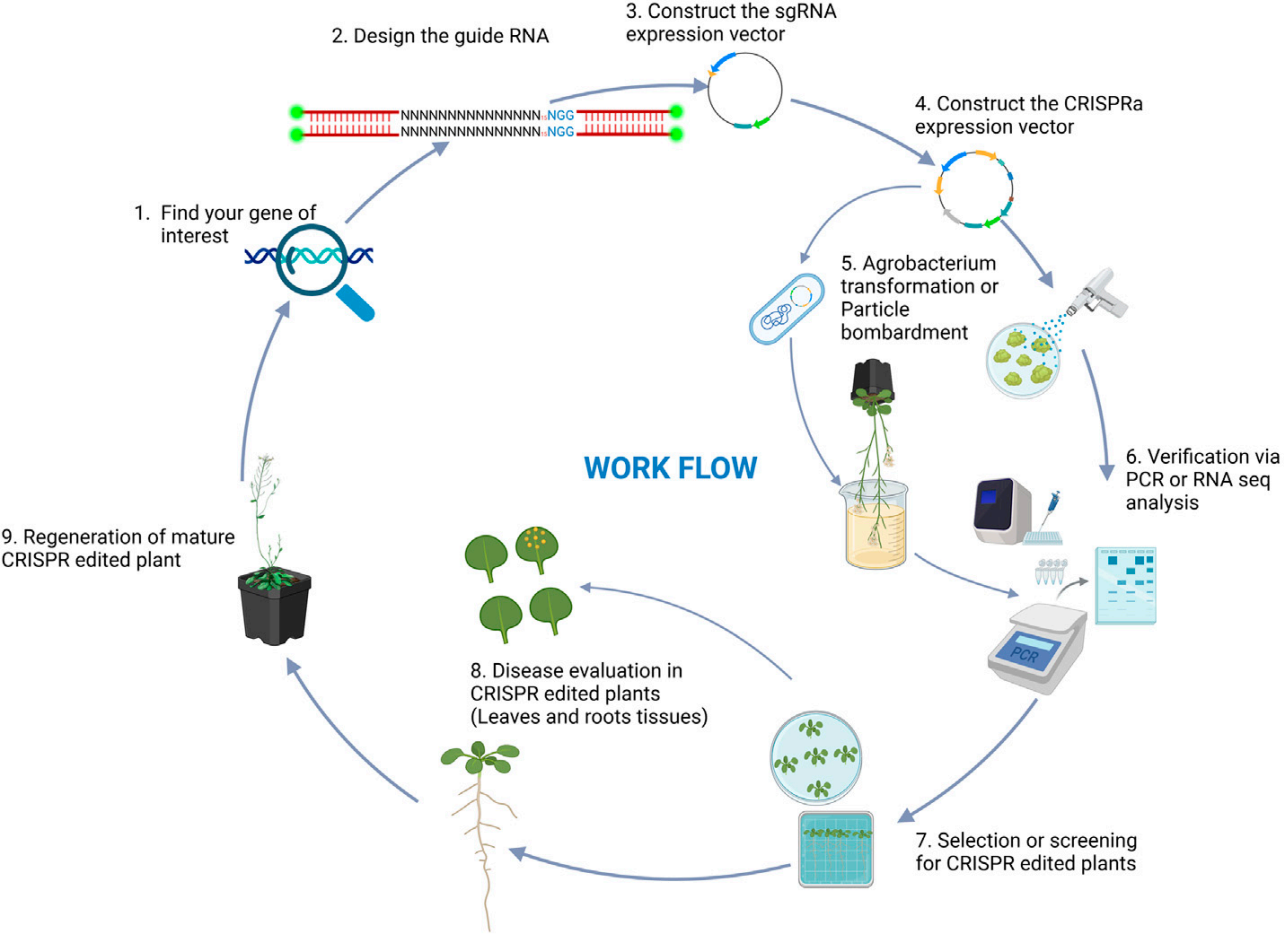
Illustration of the CRISPRa workflow in plant disease investigation.

CRISPRa, a powerful derivative of the CRISPR-Cas9 system, enables precise upregulation of target genes without introducing genomic mutations (Mori et al., 2007). Unlike conventional CRISPR approaches which are focused primarily on creating gene knockouts, CRISPRa uses dCas9 fused to a transcriptional activation domain, which selectively upregulates the expression of adjacent genes (Mori et al., 2007; McLaughlin et al., 2021). This targeted activation provides a robust tool for exploring gene function and enhancing traits related to plant resilience, including disease resistance. This mechanistic precision opens up new avenues for targeted trait improvement in crop species. Early implementations of CRISPRa employed dCas9 fused to VP64, a well-characterized and broadly active transcriptional activation domain (Pickar-Oliver and Gersbach, 2019). When guided to promoter or enhancer regions by sgRNAs, the dCas9-activator complex effectively recruits the transcriptional machinery to enhance gene expression without inducing double-stranded DNA breaks or effecting nucleotide sequence changes (Figure 3). While activation tagging, which relies on random insertional mutagenesis of the activating sequences, can result in unpredictable position effects, variable expression levels, and challenges in correlating phenotype with specific genes, CRISPRa offers a precise and reproducible approach to gene overexpression by targeting specific endogenous loci. The typical workflow for CRISPRa involves designing sgRNAs targeting promoter regions, fusing transcriptional activators to dCas9, delivering these components into plant cells, and validating gene upregulation via qPCR, RNAseq, or reporter assays (90, Figure 4). Efficient delivery systems such as *Agrobacterium*-mediated transformation, viral vectors, and emerging nanocarrier-based methods are crucial, as they directly influence transformation effectiveness, simplify cell selection, and ensure robust expression of CRISPRa components (Enright et al., 2024; Fal and Carles, 2024; Cai et al., 2023; Selma, 2024). While delivery methods are continuously being refined, these are often highly dependent on the specific cell type or organism being modified. The ability to enhance gene expression without making permanent genomic modifications makes CRISPRa a particularly promising tool for functional genomics and crop improvement (Pan et al., 2021b; Khan et al., 2025). In the field of biotic stress, CRISPRa has emerged as a powerful tool for dissecting and enhancing plant immunity through the use of targeted gene activation. By precisely upregulating genes associated with disease resistance, CRISPRa allows researchers to investigate the roles of resistance (R) and susceptibility (S) genes in plant-pathogen interactions (Mohamad Zamberi et al., 2024; Han, 2023), and this, in turn, provides a useful research platform for identifying novel genetic contributors to disease resistance and for developing resilient crop varieties.

CRISPRa can be particularly valuable for enhancing disease resistance in plants by upregulating genes involved in immune responses, stress tolerance, and growth regulation. Unlike conventional knockout approaches that target susceptibility S-genes, CRISPRa focuses on boosting the plant’s natural defense mechanisms. This is important, because the number of known disease susceptibility gene is relatively small, compared to the large number of genes known to be involved in the expression of plant immunity and defense responses. One example is the CRISPRa-mediated activation of the *PATHOGENESIS-RELATED GENE 1* (*SlPR-1*) in tomato (*Solanum lycopersicum*), which led to plants with enhanced resistance to bacterial canker caused by *Clavibacter michiganensis* subsp. *Michiganensis* (García-Murillo et al., 2023). This heightened defense is orchestrated by epigenetic reprogramming that promotes a transcriptionally active chromatin state, specifically through increased H3K4me3 deposition at the SlPR-1 promoter, which subsequently augments the plant’s salicylic acid-mediated and systemic acquired resistance (SAR) pathways upon pathogen challenge. Importantly and as noted by the authors, key agronomic characteristics were not impacted by the upregulation of *SlPR-1*. While reports on using CRISPRa to enhance disease resistance remain limited (Rivera-Toro et al., 2025; Maximiano et al., 2025; Pickar-Oliver and Gersbach, 2019), the potential of this technique for developing resilient crops is promising.

#### Additional dCas9 applications: repression and epigenetic modulation

In addition to its use in transcriptional activation, the dCas9 protein has also been repurposed for other applications such as CRISPR interference (CRISPRi) and epigenetic modification by dCas9 epi-editors (Kampmann, 2018; Enright et al., 2024; Fal and Carles, 2024; Cai et al., 2023; Selma, 2024). CRISPRi uses dCas9 fused to transcriptional repressors to inhibit gene expression by sterically blocking transcription or by recruiting repressive chromatin-modifying complexes. dCas9 epi-editors precisely target the genome to modify epigenetic marks such as H3K9me3, CpG methylation, and deacetylation (Selma, 2024; Pan et al., 2021b).

#### dCas9-based transcriptional repression (CRISPRi)

CRISPRi offers a complementary approach to epigenetic editing by effectively “turning off” gene expression without altering the underlying DNA sequence. This is achieved by fusing dCas9 with repressive effector domains, or by simply leveraging dCas9’s ability to physically block transcription when guided to a gene’s promoter or coding region. For example, CRISPRi has been widely used to silence specific genes to study their function in various biological pathways (Khan et al., 2025). This targeted gene knockdown allows researchers to efficiently investigate gene essentiality and complex regulatory networks. For example, the dCas9-SALL1-SDS3 repressor construct effectively blocks the transcription of target genes without introducing double-stranded breaks (Mohamad Zamberi et al., 2024; Han, 2023). The ability to precisely and reversibly repress gene activity makes CRISPRi an invaluable tool for functional genomics, analogous to the use of conditional mutants in conventional genetic studies.

#### dCas9-based epigenetic modulation

**FIGURE 5:**
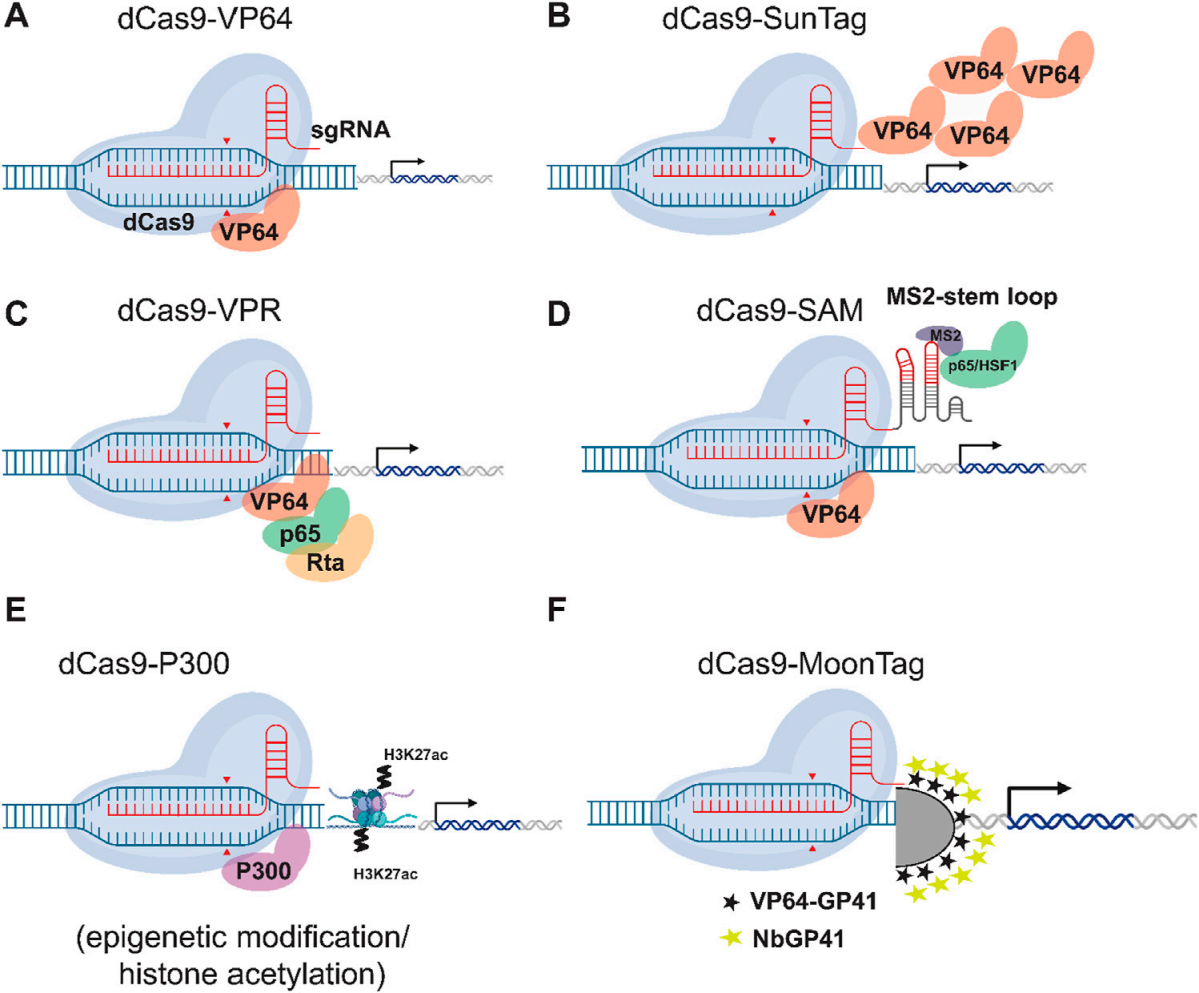
Comparison of dCas9-based Transcriptional Activation Systems. Six different strategies for CRISPR-mediated gene activation (CRISPRa) are illustrated. **(A)** dCas9-VP64: A first-generation activator in which the dCas9 protein is directly fused to the potent transcriptional activation domain VP64. **(B)** dCas9-SunTag: An amplification system in which dCas9 is fused to a repeating peptide array (GCN4). These peptides serve as a scaffold to recruit multiple copies of an antibody-VP64 fusion protein, concentrating activators at the target locus. **(C)** dCas9-VPR: A second-generation activator in which dCas9 is fused to a tripartite activator composed of three different domains: VP64, p65, and Rta, which work synergistically. **(D)** dCas9-SAM: The Synergistic Activation Mediator (SAM) system. Here, dCas9 is fused to VP64, while a modified sgRNA contains MS2 RNA aptamers. These aptamers recruit a separate protein, MS2 coat protein (MCP), which is fused to the p65 and HSF1 activation domains. **(E)** dCas9-p300: An epigenetic editing system in which dCas9 is fused to the catalytic core of the p300 histone acetyltransferase (HAT) enzyme which adds acetyl groups on histone tails (H3K27ac) to activate gene expression. **(F)** dCas9-Moontag: An amplification system, analogous to SunTag, in which dCas9 is fused to a repeating array of GP41 peptides. These peptides recruit a nanobody (NbGP41) that is fused to an activator domain like VP64. CRISPR_Cas9 BioIcons figure by Marcel Tisch and modified here using Adobe Illustrator to show a variety of different PTAs.

The versatility of dCas9 extends to targeted epigenetic modifications, offering a powerful tool for gene regulation beyond simple transcriptional activation or repression. These dCas9 epi-editors have been used to develop stable transgenics with enhanced gene expression (Liu et al., 2022). For instance, the combination of dCas9 can effect targeted DNA demethylation at a specific locus. In Arabidopsis, the human TET1 catalytic domain (TET1cd) and the improved SunTag system (further detail is shown in Figure 5) were used to alter the methylation state and subsequent activation of the FLOWERING WAGENINGEN (*FWA*) or *CACTA1* transposon within a heterochromatic locus (Fal and Carles, 2024; Shin et al., 2022; Gallego-Bartolome et al., 2018). These findings provide a foundation using CRISPR to study the roles of specific epigenetic modifications in gene regulation (Pan et al., 2021b; Shin et al., 2022).

Further examples highlight the breadth of using dCas9-based epigenetic modulation of genes. By fusing dCas9 with Arabidopsis *histone acetyltransferase 1* (*HAT1*), researchers successfully improved the expression of the AREB1/ABF2 gene (Paixão et al., 2019; de Melo et al., 2020). This epigenetic remodeling of chromatin states at specific loci led to enhanced drought tolerance in plants. In another example, researchers successfully blocked pathogen-induced gene activation in cassava by directing a zinc finger (ZF) to the effector binding elements (EBEs) within the promoter of the host susceptibility gene MeSWEET10a. This precise methylation prevented the activation normally triggered by exposure to *Xanthomonas phaseoli* pv. *manihotis*, the causal agent of cassava bacterial blight (CBB), leading to decreased disease symptoms and demonstrating a novel epigenome editing strategy for enhancing plant disease resistance (Veley et al., 2023). Due to the success of targeting a host susceptibility gene in casava and to combat cassava brown streak disease (CBSD), researchers used a dCas9-DMRcd-SunTag system to simultaneously target and methylate the promoters of two host susceptibility genes, nCBP-1 and nCBP-2, which are required by the causal Ipomoviruses (CBSV and UCBSV). While the initial results showed reduced gene expression and decreased susceptibility to CBSD, control experiments suggest that steric CRISPR interference, rather than methylation alone, was primarily responsible for the observed effect (Lin et al., 2025).

These diverse applications showcase how CRISPRa can be harnessed to achieve targeted transcriptional activation through precise chromatin remodeling, paving the way for comprehensive functional genomics studies and the development of next-generation crops with enhanced resilience. Despite its potential, CRISPRa in plants still faces challenges including developing methods for tissue-specific activation, overcoming barriers to DNA or nucleoprotein delivery, and off-target transcriptional effects. This lack of absolute specificity can result in pleiotropic effects, where activating a gene in one tissue may beneficially impact a desired trait but inadvertently cause negative effects on growth or development in another. Developing more refined, truly orthogonal inducible or tissue-specific promoters is crucial for fine-tuning CRISPRa applications. In addition, CRISPRa in plants also faces significant hurdles in overcoming delivery barriers, such as efficiently introducing CRISPRa components into diverse plant cell types and achieving stable, heritable expression. Additionally, off-target transcriptional effects can also occur, resulting from the dCas9-sgRNA complex binding to and activating (or repressing) non-target genes, potentially confounding the interpretation of experiments and possibly also leading to undesirable phenotypes.

Although CRISPRa remains relatively underexplored in plant systems, its integration with genome editing technologies offers immense potential for developing disease resistant, high yielding crops. Targeted epigenome editing via dCas9 epi-editors presents a promising transgene-free strategy to activate defense genes, paving the way for durable crop resistance. The GMO-free method would rely on CRISPR-editing components delivered in a transient fashion (e.g., via viral vectors that do not integrate into the genome or as delivery of nanoparticles containing purified proteins/RNAs), akin to Spray-Induced Gene Silencing (SIGS). In SIGS, double-stranded RNA (dsRNA) designed to target specific genes is delivered by spraying it directly onto plant surfaces (Koch et al., 2016). As more research focuses on optimizing delivery systems and improving activation efficiency, CRISPRa is poised to become an indispensable tool for sustainable agriculture and enhancing food security (Yıldırım et al., 2023; Park et al., 2024).

### Gene identification and validation: integrating CRISPRa with GWAS and multiomics technologies

While CRISPRa is a powerful tool for activating specific defense genes, its full potential in developing durable crop resistance is truly unlocked when combined with systematic gene discovery approaches. By pinpointing the most effective genetic targets through methods like Genome-Wide Association Studies (GWAS) and multiomics, CRISPRa tools can then be strategically deployed to reveal novel genetic variations, ultimately leading to enhanced plant resistance to disease. Addressing the variant-to-function (V2F) problem is critical for advancing genomics in both humans and plants (Yao et al., 2024). While GWAS can effectively identify genetic variants linked to traits or diseases (Sahito et al., 2024; Zhu et al., 2023; Zinselmeier et al., 2024), most variants occur within non-coding regions, making it difficult to discern the precise biological mechanisms responsible for phenotypic variation (Cheng et al., 2025; Lowder et al., 2015). To bridge this gap, CRISPR technology can be seamlessly integrated with advanced functional genomics approaches.

#### High-throughput screening and gene discovery

High-throughput CRISPRa screens have been used to systematically identify genes associated with human diseases (Chardon et al., 2024; Jones et al., 2022; Li et al., 2024). By using gRNA libraries combined with CRISPRa and CRISPRi, researchers can screen populations of plants for the activation of defense-related genes and uncover novel targets for crop improvement (Langner et al., 2018; Zaidi et al., 2020).

The full potential of CRISPRa for developing durable crop resistance is unlocked when combined with systematic gene discovery. Methods like STING-seq and beeSTING-seq, which integrate GWAS data with massively parallel CRISPR screens and single-cell sequencing, have been used to systematically discover target genes in mammalian systems (Morris et al., 2023). These high-throughput functional genomics strategies, combining both GWAS and CRISPR, can also be applied to uncover genes, reveal novel mechanisms, and identify breeding targets important for the expression of plant disease resistance (Clark et al., 2024).

Furthermore, multiomics (integrating genomics, transcriptomics, proteomics, and metabolomics) provides a comprehensive molecular context that can enhance CRISPRa-mediated gene discovery and validation. Transcriptomic data can highlight gene expression patterns linked to resistance, while proteomic analysis can identify defense-related signaling proteins. Integrating these datasets can help prioritize candidate genes for subsequent CRISPRa pertubation, focusing on those genes implicated in the expression plant immunity. Advances in gRNA structure design can also be used in combinatorial CRISPRa approaches, accelerating the identification of gene networks involved in plant defense and biosynthesis pathways (Fontana et al., 2024).

#### Precision gene engineering: synthetic promoters and enhancer knock-ins

Recent advancements, such as synthetic promoter engineering and enhancer knock-ins, are revolutionizing plant biotechnology by offering unprecedented and precise control over gene expression (Yao et al., 2024; Tang and Zhang, 2023). These methods move beyond traditional random transgene integration, allowing targeted manipulation of a plant’s natural regulatory machinery. For instance, researchers successfully engineered MFH17, a strong, highly constitutive synthetic promoter derived from pararetroviral elements, which effectively drives high-level gene expression across both monocot and dicot plant species (Sherpa and Dey, 2024). The ability to precisely knock-in these elements into the plant genome offers enhanced control over gene expression. Recently, researchers demonstrated a powerful strategy for improving plant abiotic stress tolerance through precise knock-ins. In this approach, CRISPR-Cas9-mediated gene targeting was used to precisely insert stress-responsive *cis*-acting regulatory elements (SRCEs) into the promoter regions of candidate genes (Ke et al., 2025). This resulted in Arabidopsis plants with enhanced tolerance to drought, salt, and osmotic stress, notably without hindering normal growth, showcasing a significant step towards improved crop resilience.

#### Streamlined approaches for plant genetic engineering

Innovations such as the CRISPR-Combo system are further streamlining plant genetic engineering (Pan et al., 2023). This system enables simultaneous, orthogonal genome editing and transcriptional regulation by employing a single Cas9 protein guided by two distinct RNA architectures. This is achieved by using a sgRNA to mediate double-strand breaks for mutagenesis at one locus, while a concurrently deployed, engineered scaffold sgRNA recruits transcriptional activator complexes to a separate promoter, thereby upregulating gene expression without inducing DNA cleavage. This system enables speed breeding of transgene-free, genome-edited Arabidopsis plants and also substantially enhances hormone-free rice regeneration, hence increasing the pool of regenerated plantlets available for screening heritable, targeted mutations (Pan et al., 2023; Haber et al., 2024). This innovative approach allows for the simultaneous modulation of both DNA and RNA, accelerating trait stacking and improving crop resilience by streamlining the enhancement of desired traits while suppressing unwanted pathways (Gardner et al., 2025; Jaegle et al., 2025). The comprehensive all-in-one CRISPR toolbox (Cheng et al., 2025) further simplifies guide RNA library cloning, making large-scale genetic screens more time and cost-efficient. This toolbox has been successfully used to engineer herbicide resistance in rice by employing PAM-less CRISPR-Cas9 base editors to target the acetolactate synthase (*OsALS*) gene, enabling comprehensive coverage of known resistance-associated regions and the discovery of novel herbicide-resistant alleles (Clark et al., 2024). Similar experiments conducted in protoplasts hold promise for discovering and evaluating genes related to disease resistance, particularly for testing PTAs in plant immunity (Casas-Mollano et al., 2023; Mukundan et al., 2025; Sychla et al., 2022).

#### Translating technologies to crop improvement

Integrating CRISPRa with GWAS and multiomics, combined with advancements in precise gene editing, holds immense potential for crop improvement. Numerous GWAS studies have identified disease-resistant regions in the genomes of various crop plants (Gangurde et al., 2022; Gardner et al., 2025; Jaegle et al., 2025; Sahito et al., 2024; Zhu et al., 2023). These integrated approaches can now accelerate the discovery, characterization, and modification of genes within these identified regions. A recent study proposed using high-quality GWAS data for grain total weight traits and applying CRISPR to modulate expression (Jamil et al., 2025). As applied to disease resistance, this approach could mean accelerating the development of broad-spectrum resistance in wheat to devastating fungal diseases like Fusarium head blight, rust and powdery mildew, significantly reducing yield losses. Similarly, in rice, the precise knock-in of regulatory elements could enhance tolerance to environmental stressors, such as specific soil pathogens or extreme temperatures, leading to more stable and higher yields in vulnerable regions. By identifying and precisely modulating the expression of key defense genes or stress response pathways, these sophisticated molecular tools can pave the way for developing more resilient and productive crops.

### Programmable transcriptional activators in plant immunity

PTAs are engineered proteins designed to specifically bind to DNA sequences and activate the transcription of target genes (Rivera-Toro et al., 2025; Maximiano et al., 2025; Jamil et al., 2025). While earlier PTAs relied on platforms like zinc-finger transcription factors (ZF-TFs) or transcription activator-like effector nucleases (TALE-TFs), recent advancements, particularly with CRISPR-based PTAs, offer enhanced specificity, greater versatility and improved experimental turn-around time (Zinselmeier et al., 2024).

In the context of plant immunity, PTAs provide a powerful tool for manipulating gene expression and enhancing disease resistance. By carefully designing PTAs to target the promoter regions of resistance genes, more precise control of expression levels can be achieved, allowing fine-tuning of the plant defense response. Early CRISPR-based PTAs often utilized a dCas9 fused to the VP64 transcriptional activation domain (Li et al., 2020). VP64 is a synthetic transcriptional activator composed of four tandem repeats of the minimal activation domain from the Herpes Simplex Virus (HSV) protein VP16. The dCas9-VP64 fusion successfully enhanced endogenous genes in Arabidopsis, rice, and tobacco (Lowder et al., 2015). However, more sophisticated second generation PTAs have since emerged, incorporating various potent activation domains like the EDLL domain or the VPR activator, which can significantly boost gene expression levels (Maximiano et al., 2025; Zaidi et al., 2020; Morris et al., 2023). The plant-derived EDLL motif, a compact and potent transcriptional activation domain from the AP2/ERF factor family, has been effectively harnessed as an effector domain for dCas9-based synthetic activators in plant systems. The VPR system is a potent, tripartite transactivation module created by fusing three distinct activator domains-VP64, p65, and Rta-into a single protein (Pan et al., 2021a). A parallel strategy for developing advanced PTAs is the Synergistic Activation Mediator (SAM) system, which achieves potent transactivation by integrating a dCas9-VP64 fusion with a modified sgRNA harboring MS2 aptamers; these aptamers then recruit a separate MS2-p65-HSF1 effector protein for synergistic gene activation (Ding et al., 2022). Similarly, the SunTag system achieves this by fusing the dCas9 protein to multiple copies of the GCN4 peptide, which then serve as an anchor to recruit numerous antibody-activator fusions (scFv-VP64), thereby amplifying the transcriptional signal at the target gene (Pan et al., 2021a; Papikian et al., 2019). The MoonTag system was developed as a programmable transcriptional activator to overcome the poor expression and stability of the SunTag system’s scFv antibody component in plants, replacing it with a more robust llama nanobody (NbGP41) and its corresponding GP41 peptide epitope. In this system, a dCas9 protein fused to tandem GP41 repeats recruits multiple copies of an NbGP41-VP64 activator protein, resulting in a well-tolerated tool that powerfully and efficiently activates genes in diverse plant species including *Setaria*, *Arabidopsis*, and tomato (Casas-Mollano et al., 2023; Zinselmeier et al., 2024). For instance, CRISPR-based PTAs have been shown to lead to a 100- to 2000-plus fold increase in gene expression in tobacco, specifically when targeting endogenous promoters (Garcia-Perez et al., 2022). In this latter study, carried out in tobacco, activation domain dCas9:EDLL (Tiwari et al., 2012), was fused to the copper-responsive factor CUP2, which was then able to induce the genes dihydroflavonol 4-reductase (*DFR)* (2600-fold) and phenylalanine ammonia-lyase *PAL2* (245-fold), respectively, following the application of 5 mM CuSO_4_. Because high mRNA levels do not guarantee a proportional increase in protein due to cellular regulation, Western analysis should be used as a validation step in future CRISPRa studies to confirm the impact of transcriptional activation on the accumulation of the intended functional protein.

The application of PTAs to enhance plant immunity, especially in combination with CRISPRa technologies, holds immense promise. Combinations of PTAs fused to dCas9 in *Nicotiana benthamiana* have achieved extremely high levels of transcriptional activation (Zaidi et al., 2020). This approach is also useful for producing commercially important metabolites. PTAs have been shown to be effective in Arabidopsis and rice protoplasts (Li et al., 2017). PTAs like dCas9-VP64 and dCas9-TV can effectively upregulate gene expression in grapes, as demonstrated by the successful activation of genes like *UDP-Glucose: Flavonoid 3-O-Glucosyltransferase* (*UFGT*) and *C-repeat Binding Factor 4* (*CBF4*), leading to potentially beneficial traits such as increased cold tolerance (Ren et al., 2022). PTAs also provide a valuable tool for studying the dynamic interplay of genes involved in plant defense. By precisely controlling the expression levels of specific genes, researchers can dissect their individual roles in complex signaling pathways and identify key regulatory nodes. This knowledge can then inform the development of targeted strategies for enhancing disease resistance in crops. Looking ahead, the evolution of PTA technology promises even more sophisticated control over plant immune networks. Future developments will likely focus on multiplexed activation, where a single CRISPR-based system uses an array of guide RNAs to simultaneously upregulate multiple defense-related genes, thereby engineering complex resistance traits or entire signaling pathways at once. Tool kits are available to help researchers design and implement multiplexed activation (Pan et al., 2021a; Cheng et al., 2025). Furthermore, integrating PTAs with inducible systems, such as those responsive to light, metals (like the copper example above), chemicals, or specific pathogen-derived molecules, will enable precise spatiotemporal regulation (Rahman et al., 2022; Zhang et al., 2025). This would allow for the activation of immunity genes only at the specific time and location of an infection, maximizing defense effectiveness while minimizing potential fitness costs to the plant. Figure 5 gives a summary of some first- and second-generation PTAs mentioned in this work.

#### Achieving overexpression without GMOs

The ability to enhance crop traits without inserting foreign DNA has become an important goal in plant biotechnology, reflecting both the regulatory landscape and the public trust of gene-edited foods versus genetically modified organisms (GMOs). GMOs are plants or animals modified through methods that do not occur naturally, often involving the insertion of genes from other species to introduce traits such as pest resistance or drought tolerance (Holst-Jensen et al., 2012; Waigmann et al., 2012). In 2018, the U.S. Department of Agriculture (USDA) announced that genome-edited plants indistinguishable from those developed through traditional breeding methods would not be subject to regulation, highlighting the distinction between conventional GMOs and gene-edited organisms (Grossman, 2019; Menz et al., 2020). Notably, CRISPR-based technologies can produce gene-edited plants with enhanced disease resistance that are considered transgene-free, since no foreign DNA is integrated into the final product. This has been demonstrated in studies involving basil (Zhang et al., 2021) and wheat (Li et al., 2022), using transient CRISPR-gene editing vectors.

CRISPR technology holds significant promise for enhancing crop resilience against biotic and abiotic stresses, improving yield potential, and achieving these advancements with minimal environmental impact. Despite these benefits and the scientific and regulatory distinctions between gene-edited crops and conventional GMOs, public perception and acceptance remains a significant barrier. This challenge is particularly pronounced in regions such as the European Union, where public opposition to GMOs has been persistent and regulatory frameworks remain stringent (Kato-Nitta et al., 2023).

Several CRISPR-edited crops developed using traditional CRISPR-Cas9 or similar systems for gene knockout or precise gene edits have been approved for commercialization (Table 1). Interestingly, while these crops were developed using gene-editing tools like CRISPR-Cas9, they do not contain integrated foreign genes in their genomes. This distinction is critical under certain regulatory frameworks in which gene-edited plants are not classified as GMOs, potentially easing regulatory barriers and public concerns.

**TABLE 1:** Examples of Commercially Approved and/or Released Gene-Edited Crops.

Crop	Developer/Institution	Modified trait and genetic target	Approval/Release (Year, Jurisdiction)	References
Non-Browning Mushrooms	Yinong Yang (Penn State University)	Reduced browning by knocking out the polyphenol oxidase (PPO) gene	2016, U.S. (USDA approval)	Heynes et al. (2022)
Waxy Corn	Corteva (formerly DuPont Pioneer)	Altered starch composition (high amylopectin) by modifying the waxy gene	2016, U.S. (USDA approval)	Ricroch (2019)
High-Fiber Wheat	Calyxt (using TALENs)	Increased dietary fiber content (up to 3x) in the flour	2018, U.S. (USDA approved; awaiting commercialization)	Ricroch (2019)
High-Oleic Soybean (Calyno™ oil)	Calyxt (using TALENs)	Healthier oil profile with high oleic acid and reduced saturated fats; extended fry life	2019, U.S. (Commercial release)	Waltz (2018)
Sulfonylurea-Resistant Canola	Cibus	Herbicide resistance by modifying the acetolactate synthase (ALS) gene	2020U.S.	Subedi et al. (2020)
High-GABA Tomatoes	Sanatech Seed	Enhanced gamma-aminobutyric acid (GABA) content for potential health benefits	2021, Japan	Waltz (2022)
Drought-Tolerant Soybeans	Benson Hill	Enhanced drought resilience by improving water use efficiency	2022U.S.	Camerlengo et al. (2022)
Purple/Less Bitter Mustard Greens	Pairwise	Reduced pungency/bitterness to improve flavor, making them more like lettuce	2023, U.S. (Limited foodservice release)	Ricroch (2019)
Lettuce	GreenVenus, Llc	Inactivating the polyphenol oxidase (PPO) gene to prevent enzymatic browning, Shelf life	2023	Ricroch (2019)
High-Antioxidant Purple Tomato	Norfolk Plant Sciences	Increased anthocyanin (antioxidant) levels by introducing two genes from snapdragon flowers	2024, U.S. (Seeds for sale to home gardeners)	Martin and Butelli (2025)
Disease-Resistant Rice	China Agricultural University	Developed varieties with enhanced resistance to major diseases like bacterial blight and rice blast	2024, China (Biosafety certificate granted)	Liang et al. (2025)

However, the application of CRISPRa specifically in commercial agriculture remains largely unexplored, with current research focusing on enhancing disease resistance and other agronomically important traits (García-Murillo et al., 2023). This cautious pace of commercialization stems from several factors, including the technical challenges of efficiently delivering the large CRISPRa protein complexes into plant cells, ongoing regulatory uncertainty distinct from that for gene knockouts, and the need to validate stable and predictable gene activation across diverse field conditions. Additionally, progress will be influenced by the discovery of key regulatory elements (such as upstream regulatory regions) which can then be edited by CRISPR (Si et al., 2020; Xiang and Dong, 2025).

Public perception of gene-edited crops continues to be mixed, largely stemming from a persistent confusion with traditional GMOs (Frisio and Ventura, 2019; Thornton, 2025; Vindigni et al., 2022). This is reflected in consumer data; for example, while the cited study on willingness-to-pay for CRISPR-edited rice showed varied acceptance across countries, other consumer surveys consistently find that public support increases when the specific benefits, such as enhanced nutrition or improved disease resistance leading to lower pesticide use, are clearly explained (Vasquez et al., 2022). However, initial skepticism often remains high without this context.

This knowledge gap highlights the pivotal role of proactive science communication and educational outreach. To build public trust, it is essential for the scientific community and industry stakeholders to transparently explain the distinction between transgene-free gene editing and conventional genetic modification. Clear, accessible information about the technology’s precision, safety assessments, and tangible benefits, as well as any risks, can empower consumers to make informed decisions rather than relying on outdated perceptions of GMOs. As regulatory frameworks continue to evolve, fostering an informed public dialogue through these outreach efforts will be as critical as the scientific advancements themselves in guiding policy decisions and shaping the future of CRISPR-based crop improvements for sustainable agriculture.

While significant consumer and market resistance has historically blocked GMOs in U.S. staple crops like wheat and barley, the widespread adoption of transgenic corn and soybeans reflects a permissive regulatory trend that is mirrored in other key agricultural nations. For example, Brazil’s regulatory agency (CTNBio) established early on that crops with transgene-free edits are not considered GMOs, streamlining approvals for products like high-yield sugarcane (Segretin et al., 2025). Similarly, India’s government has exempted certain categories of gene-edited plants from its stringent GMO regulations to accelerate crop improvement (Sankaranarayanan, 2024). Most notably, China has recently shifted its policy to create a clearer, more efficient approval pathway for gene-edited organisms, granting biosafety certificates for staple crops like wheat and rice in 2024 to bolster its food security goals (Liang et al., 2025). This contrasts with more cautious regions like the European Union, creating a complex and evolving global regulatory landscape for these technologies.

### High-throughput phenotyping for mutant identification

Researchers can screen for genes that confer enhanced disease resistance by generating CRISPRa guide RNA (gRNA) libraries that target a wide array of genes or, potentially, all genes (Pan et al., 2021a). This activation-based strategy is powerfully complemented by high-throughput phenotyping, as it enables large-scale GOF screens. Unlike traditional mutagenesis, which identifies necessary genes by observing the negative effects of gene knock out, a CRISPRa screen can reveal beneficial traits that emerge when a specific gene’s expression is increased. Advanced, automated phenotyping is therefore essential for detecting these often subtle improvements in disease resistance or stress tolerance across vast plant populations, allowing researchers to efficiently pinpoint the specific gene activations that enhance plant fitness and accelerate crop improvement (Arshad et al., 2025; Ninomiya, 2022).

Screening such large, CRISPRa-activated populations benefits greatly from advances in high-throughput, automated phenotyping. A suite of non-destructive technologies enables the early detection of stress and disease symptoms. These include 3D laser scanning along with hyperspectral, thermal, RGB, Near-Infrared (NIR), and fluorescence imaging, which is used to assess photosynthetic efficiency (Almoujahed et al., 2025; Grishina et al., 2024; Kurumayya, 2025). Analysis of the data from these imaging tools can reveal subtle physiological changes that are not visible to the naked eye (Bao et al., 2024; Bauriegel and Herppich, 2014). For instance, while a fungal pathogen like *Fusarium graminearum* eventually causes visible bleaching of infected tissue, fluorescence imaging can detect impacts on photosynthetic metabolism at much earlier stages of disease, serving as an excellent phenotypic assay (Bushnell et al., 2010). In addition, these experiments can be performed along a time-course which can allow researchers to capture the disease process from start to finish, enabling higher quality and enriched phenotyping data.

The high-throughput methods used to analyze these populations are themselves highly advanced. While many foundational studies have used these techniques to screen traditional mutant libraries for traits like disease resistance (Almoujahed et al., 2025; Femenias et al., 2020; Leiva et al., 2022; Mahlein et al., 2019), the same platforms, such as robotic ground sampling or remote sensing with a drone, are directly transferable to screening large CRISPRa-activated populations. A prime example of a CRISPRa-specific application would be the activation screening of a library targeting thousands of plant transcription factor genes. This population could then be challenged with a fungal pathogen, and automated hyperspectral imaging could be deployed to detect subtle differences non-destructively in disease progression, thereby identifying which specific transcription factors orchestrate a more effective defense response when overexpressed.

These techniques have also proven successful in post-harvest analysis, such as scanning harvested grain for the presence of the mycotoxin deoxynivalenol (Su et al., 2021). Hyperspectral scanning phenotyping combined with GWAS methods has been used to map regions of the wheat genome that impact deoxynivalenol accumulation in the resulting grain (Concepcion et al., 2024). In addition, machine learning and AI will play a crucial role in analyzing phenotypic data, identifying patterns, and predicting trends, thus enhancing the efficiency and precision of identifying beneficial gene activations (Jiang and Li, 2020; Kaya, 2025; Kundu et al., 2024; Maraveas, 2024).

The integration of CRISPRa technology with these advanced screening methods provides a powerful approach to dissect gene function and assign positive traits to specific overexpressed genes, paving the way for significant advancements in breeding for resistance. This multidisciplinary approach is essential for managing the complex data and experimental demands of screening large populations for disease resistance.

## Conclusion and prospectives

### A new paradigm for enhancing plant defense

CRISPRa offers unprecedented control over gene expression and permits new types of genetic studies that may reveal novel genetic and phenotypic variation. The focus of this review has been on plant disease resistance, but the impact of this technology for crop improvement will be broad and substantial. By enabling the controlled upregulation of specific endogenous genes involved in immunity, CRISPRa offers a promising strategy to enhance disease resilience in crops. Its precision and ability to modulate gene expression levels make it especially valuable for fine-tuning defense pathways without compromising plant growth and development. This balance is critical, as excessive activation of immune responses can lead to detrimental trade-offs in plant fitness (Giolai and Laine, 2024; He et al., 2022). Its targeted nature and ability to fine-tune endogenous gene expression allows for much greater precision in manipulating plant defense responses.

### Synergistic integration with modern genomics

The power of CRISPRa is amplified when integrated with other functional genomics approaches. Combining CRISPRa with tools like GWAS and multiomics analyses allows researchers to systematically investigate the functional consequences of genome-wide genetic variation and its impact on plant immunity, which often relies on quantitative genetic factors (Jamil et al., 2025; van Schie and Takken, 2014).

Furthermore, a particularly powerful strategy involves combining CRISPRa with standard CRISPR-based gene editing. This allows researchers to simultaneously activate key resistance genes while disabling genes whose presence in plants increases vulnerability to infection and disease development (susceptibility genes), thereby creating a synergistic effect that could produce crops with exceptionally robust defense responses. However, despite these advantages, several challenges remain. Efficient and tissue-specific delivery of dCas9-based effectors, along with the identification of suitable promoters to ensure consistent activation across plant species and tissues, represent significant hurdles that must be overcome in order to fully implement the potential of CRISPRa in agriculture.

### Future prospects and the path to application

While gene activation strategies have been greatly underutilized in plant disease studies, compared to their impact in bacterial and mammalian cells (Bikard et al., 2013; Casas-Mollano et al., 2023), recent methodological advancements are poised to accelerate progress. A significant step toward commercial application involves moving away from reliance on transgenic components. Compared to classical overexpression using constitutive promoters, CRISPRa offers a significant advantage in its ability to fine-tune gene expression, allowing for more precise control of transcription levels and minimizing unintended phenotypic imbalances. Although CRISPRa currently requires the introduction of CRISPR components for gene activation, emerging strategies are enabling gene overexpression without permanent integration of foreign DNA. One such strategy involves editing targeting regulatory elements upstream of native genes to upregulate genes.

As this technology matures, we can expect an increase in the application of CRISPRa for enhancing plant immunity and other complex traits. The ability to precisely activate specific genes holds the key to unlocking new possibilities for crop improvement and ensuring global food security. This technology holds transformative potential not only for enhancing disease resistance but also for traits such as abiotic stress tolerance, yield improvement, and nutrient use efficiency making it a cornerstone for next-generation crop breeding.
